# Hip Hop Stroke: Study Protocol for a Randomized Controlled Trial to Address Stroke Literacy

**DOI:** 10.4172/2167-0870.1000242

**Published:** 2015-10-23

**Authors:** Olajide Williams, Ellyn Leighton-Herrmann, Alexandra DeSorbo, Mindy Hecht, Monique Hedmann, Saima Huq, William Gerin, Vernon Chinchilli, Gbenga Ogedegbe, James Noble

**Affiliations:** 1 Department of Neurology, Columbia University Medical Center, USA; 2 Department of Biobehavioral Health, The Pennsylvania State University, USA; 3 Department of Public Health Sciences, The Pennsylvania State University College of Medicine, USA; 4 Department of Population Health, NYU School of Medicine, USA

**Keywords:** Stroke, Health disparities, Randomized trial, Stroke health education, Child-mediated communication, Health communication

## Abstract

**Objective:**

Stroke is the fifth leading cause of death and the leading cause of serious long-term adult disability in the US. Acute stroke treatments with intravenous thrombolysis and endovascular therapy are proven to reduce disability, however a critical limitation on their effectiveness is the narrow time window for administration, which is 4.5 hours and 6 hours respectively from the onset of symptoms. Our overarching goal is to reduce pre-hospital delays to acute stroke treatments in economically disadvantaged minority communities where the greatest delays exist, using Hip Hop Stroke.

**Methods:**

Hip Hop Stroke (HHS) is a school-based, child-mediated, culturally-tailored stroke communication multimedia intervention developed using validated models of behavior change and designed to improve stroke literacy (knowledge of stroke symptoms, the urgent need to call 911, and prevention measures) of 4^th^, 5^th^ and 6^th^ grade students and their parents residing in poor urban communities. Children in the intervention arm will receive the HHS intervention, while those in the attentional control arm will receive standardized nutrition education based on the USDA's MyPyramid program. Children will be trained and motivated to share stroke information with their parents or other adult caregiver. Both children and parents will complete a stroke knowledge assessment at baseline, immediately following the program, and at 3-months post-program. The primary outcome is the effect of the child mediation on parental stroke literacy.

**Conclusion:**

Stroke literate children, a captive audience in school systems, may represent a viable channel for spreading stroke information into households of poor urban communities where mass media stroke campaigns have shown the lowest penetration. These children may also call 911 when witnessing a stroke in their homes or communities. The HHS program may highlight the potential role of children in the chain of stroke recovery as a strategy for reducing prehospital delays to acute stroke treatment.

## Introduction

Stroke is the fifth leading cause of death and the leading cause of serious long-term adult disability in the US, and it has a 2-fold greater incidence in Blacks when compared to the majority of Americans [1–6]. An estimated 795,000 new and recurrent strokes occur in the US each year [7]. Stroke remains the second leading cause of death among Blacks, and Blacks are at least twice as likely to die from stroke [1–3]. The economic burden is immense, with the combined direct and indirect cost of stroke totaling more than $33 billion in 2011 [7].

Intravenous thrombolytic therapy with tissue plasminogen activator (t-PA) is a proven treatment for acute ischemic stroke patients up to 4.5 hours after symptom onset [8–11]. Following an ischemic stroke event, the administration of t-PA can improve the patient’s odds of having minimal to zero disability at 3 months by 31%–50% [12]. Approximately 7% of diagnosed ischemic stroke events currently receive t-PA therapy [13], due mostly to the public’s lack of knowledge about the treatment and the inability to identify and respond appropriately to stroke symptoms when they occur [14,15]. If the rate of all ischemic stroke patients receiving t-PA increased to only 10%, the realized annual cost savings to taxpayers would be more than $45 million [16].

A critical limitation to the effectiveness of treatment with t-PA is the narrow treatment window. For the majority of stroke patients, this window is 3–4.5 hours from the onset of symptoms. However, recent studies support the extension of this window to 6 hours for patients who are eligible for endovascular therapies [17]. It is critical that a person experiencing or witnessing a stroke be able to identify the symptoms and know to immediately to call 911. It has been estimated that interventions designed to educate patients to seek treatment sooner when a stroke occurs may increase thrombolysis rates to 57%, if emergency medical system response times and in-hospital response times are optimized [18].

In 2002, National Institute of Neurological Disorders and Stroke (NINDS) targeted expediency of stroke recognition to increase t-PA use, with a goal of 70% of stroke patients arriving at the hospital within three hours by 2013 [19]. Unfortunately, this goal is not close to being met, with recent data suggesting that only 22% of patients typically arrive within 3 hours of last known well [13]. New and effective interventions that improve the behavioral response to the acute stroke situation in a cost-effective manner are necessary to meet these goals, given the strong relationship between early hospital arrival and acute stroke treatment [20].

### Bridging a health literacy gap: Child-mediated health communication

Children can serve as community health educators: A growing body of literature supports the potential utility of children as conduits of health information for their elders and communities [21–25], yet young people are often overlooked as sources of important health information. One of the few attempts to engage children in health prevention was an asthma education program “Open Airways for Schools,” which positively influenced parental health behaviors [21]. Given the low rate of parental attendance at school sessions, investigators held six sessions for students aged 8 to 11 years with asthma at school, and gave the children homework assignments to complete with their parents at home to teach the parents and build support for children’s self-management efforts. Another study which delivered hypertension education to school children showed that children improved parents’ knowledge about hypertension and increased the likelihood that the parents will consult their physician about their blood pressure [25].

Expanding on these strategies, NINDS suggested that stroke-educated children could improve their own stroke risk and possibly educate their parents and relatives about stroke [18]. It has been found that up to 45% of public stroke knowledge is derived from family and friends [26], suggesting that children may be an underutilized means of stroke education in their homes and communities. In response to this health education paradigm, we created the Hip Hop Stroke program.

### Innovation

We aim to reduce the aforementioned pre-hospital delays using Hip Hop Stroke (HHS), [27] a novel behavioral intervention to improve symptom recognition and response in a high-risk, minority, economically disadvantaged population. Despite the abundance of available stroke education materials, studies continue to reveal severe deficiencies in stroke literacy (i.e., knowledge of symptoms, urgent action, and prevention measures) [28]. Expensive mass media stroke education campaigns are not sustainable for this purpose, particularly in economically disadvantaged populations, which they are often not tailored to penetrate. As such, this study intervenes in elementary schools with 4^th^ to 6^th^ grade children, ages 9 to 11. HHS delivers stroke education around key program centerpieces comprised of rap songs and two animated musical cartoons that incorporate stroke knowledge. This multimedia intervention teaches the five cardinal stroke symptoms and the correct course of action when they occur. It also highlights time-dependent acute stroke treatment with “clot busters” and the potential therapeutic benefit of early hospital arrival. The intent of the intervention is that the children will then educate their parents and community members with the stroke knowledge they have gained.

**Figure 1:**
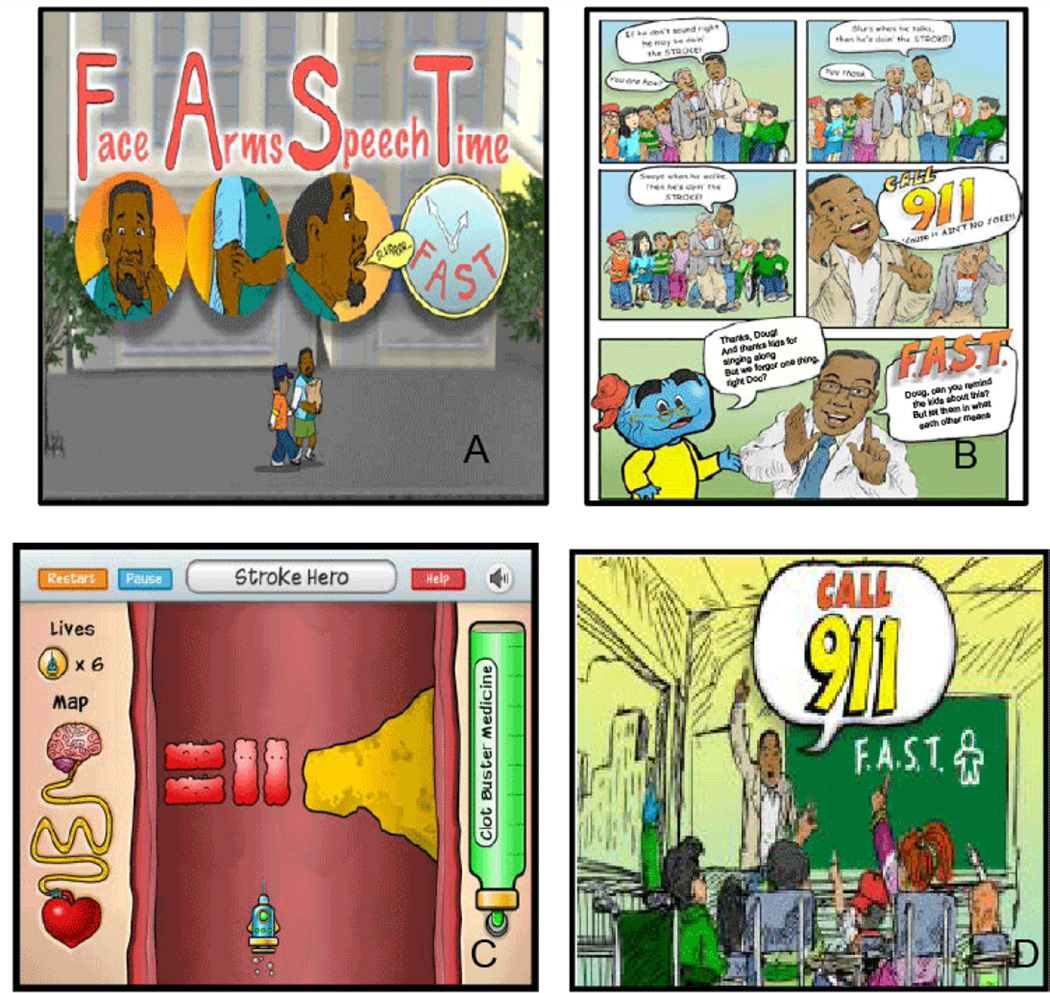
Sample Hip Hop Stroke media. A: “Keep Your Brain Healthy”: Animated feature showing the FAST mnemonic; B: Hip Hop Stroke Comic book; C: “Clotbuster” video game; D: “Stroke Ain’t No Joke”: Animated feature.

In preliminary research, we found that children aged 9–11 years can rapidly learn and retain the information well for at least 15 months [29]. Our recent pilot study showed that 74% of children in the pilot (N=182) communicated the material to a parent, and that this communication significantly improved the parent’s stroke literacy [30]. Having demonstrated the efficacy of the HHS intervention in a smaller pilot sample, the current study will use a randomized controlled trial of this health intervention to evaluate the effect of the HHS intervention on: (1) children’s ability to learn and retain the information, (2) the likelihood that children participating in HHS can and will engage their parents and teach them the warning signs and symptoms of stroke and the appropriate “911” response to these symptoms, and (3) parents’ ability to learn and retain the information (Figure 1).

**Figure 2:**
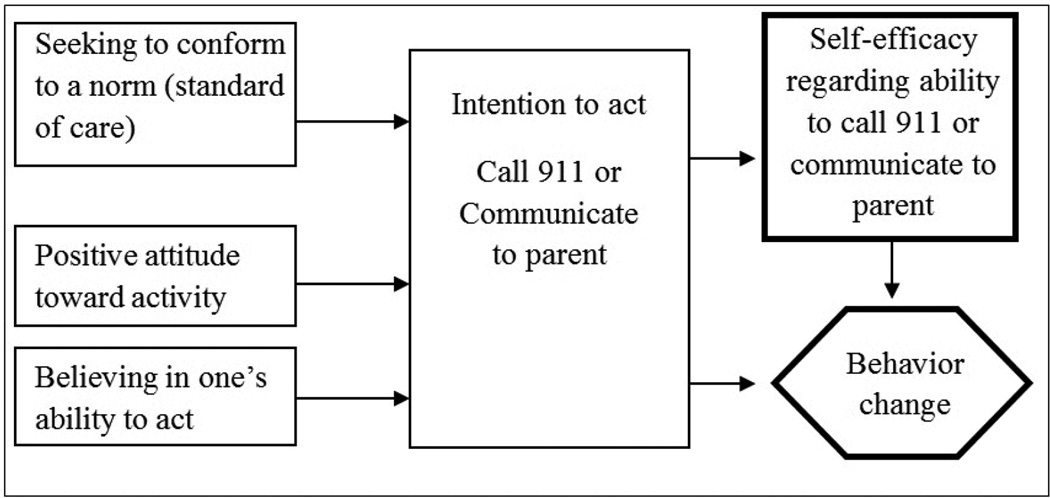
Theoretical underpinnings of Hip Hop Stroke.

HHS is based on two models that have been demonstrated as important predictors of behavior change. Theory of Reasoned Action suggests that a series of related cognitive constructs operate to produce an intention to act, which is clearly a precursor to the desired outcome, namely engaging in the act (i.e., making use of the stroke information as part of standard practice) [31]. The second is Self-Efficacy Theory, which posits that control over one’s outcomes produces a sense of mastery for those behaviors (in our case recognizing stroke and calling 911, and communicating this information to parents); and that increased self-efficacy predicts increased motivation to engage in the desired behaviors, as well as a demonstrated increase in the behavior itself (Figure 2).

## Methods

### Study design overview

This protocol is for Hip Hop Stroke (HHS), a school-based intervention aimed at educating 4^th^, 5^th^ and 6^th^ grade students about stroke symptoms, urgent “911” response and prevention measures. We term this stroke literacy. The intervention will be delivered in a school auditorium or gym setting, using an innovative, modular, multi-media program that contains embedded cartoons and music teaching stroke recognition, appropriate action, availability of time dependent treatment, and prevention, all visually presented via Microsoft PowerPoint with a projector in conjunction with a pair of facilitators consistent throughout the study and whose performance is monitored internally through quality assurance measures. In addition, students will be given specific mediating tools for parental learning. These are home-based activities to complete with their parents to increase parental stroke literacy. There will be a total of four days of programming per school with one hour of instruction on each day: an initial 3-day program and a 1-day booster session three months later. This study has received ethics approval from the Columbia University Medical Center Institutional Review Board and the New York City Department of Education Institutional Review Board.

### Setting

This study will be conducted in 22 New York City public schools (11 intervention; 11 control) in the boroughs of Brooklyn, Queens and the Bronx. Administrative leadership in schools and school districts has welcomed this program and the setting has proved to be ideal for the delivery of the intervention in our pilot studies. School officials are motivated to provide the support for several reasons, including the requirement to implement health education curriculums with strong primary prevention in their schools, which has been incorporated into this program.

### Recruitment and randomization

Randomization will occur at the school level, rather than by child or classroom, to ensure minimal cross-contamination between intervention and control groups. During the study period, we anticipate that parents in the control condition will likely get stroke information only from existing public education efforts in the form of television or radio outreach.

#### School and participant eligibility

In order to be eligible, participating schools must have a high percentage of Black students (>15%) and students who receive free or reduced-fare lunches (>50%). These two criteria will help ensure that we reach high need populations, with low levels of health literacy and stroke knowledge and high levels of stroke incidence. Additionally, participating schools must have a low percentage of English Language Learners (<20%) to ensure that both the students and their parents are able to thoroughly comprehend this English-language program. Participants will include 4^th^, 5^th^ and 6^th^ grade students (ages 9–12) and their parents. In order to be eligible, participants must have sufficient English language skills.

#### School recrtuitment

Twenty-two New York City public schools with 4^th^, 5^th^ and/or 6^th^ grades, located in the boroughs of the Bronx, Queens and Brooklyn will be invited to participate in the study. School principals identified by school district superintendents, department of education health directors and local relationships that meet the eligibility criteria will be contacted by phone or email. Research staff will initiate contact with the school and give a full description of the study. A meeting will be scheduled with research staff and the school administration to show a ten-minute explanatory presentation about the HHS curriculum and program objectives. Participating schools will receive a $1,000 grant as a thank you for providing their time and space as well as assisting with parental recruitment. Once the principal has agreed for his/her school to participate, he/she will sign a participation agreement form outlining the school’s responsibility and terms of participation.

#### Student recruitment

Once the school agrees to serve as a site, all the children in the 4^th^, 5^th^ and/or 6^th^ grades are included in the program. Students will be introduced to the program at a brief presentation led by one health educator and/or research assistant (RA), in which each child will be given a study information sheet and consent form for their parents. Any student who returns a signed consent form from a parent or caregiver receives a prize, whether the parent agrees to participate or not. We estimate that approximately 3,500 students will be in the final sample.

#### Parent recruitment

Prior to program commencement, parents will be recruited via two methods: in-person at school events, such as orientation and parent/teacher conferences and via the student-based presentation discussed above, in which consent forms are sent home for completion. To maintain statistical independence, only one adult from each family will be interviewed. To assess the primary outcome of parental knowledge, we will recruit at least 860 parents across 22 schools.

### Informed consent and assent

Informed consent will be obtained from parents for the enrollment of their child and themselves into the study. If recruited in-person, the RA will give the parent a consent form, describing the study and the details of participation. The RA will tell the parents what their informed consent entails, including the purpose of the study, the risks and benefits of participation, the voluntary nature of their participation, and the fact that participation does not affect their child’s grades in school. If recruited via the student session, the parent will complete the consent form and return it with their child. With this method, they are provided with study contact information, so they may ask questions before consenting. Student assent will be obtained from each participating child on Day 1 of the program. Students are also informed that all testing will have no impact on their grades.

### Retention

Parents will be mailed a small token of appreciation after each survey. Prior to the delayed follow-up, parents will be mailed a greeting card, thanking them and reminding them of their participation in our study. At baseline, parents will be asked to provide the names and phone numbers of three close contacts who will know how to reach them, in the event that their address and/or telephone number changes (i.e., retention contacts).

### Study conditions

#### Implementation overview

Health educators certified by six hours of HHS training which includes live observation (and one hour of annual retraining) will conduct both the intervention and control programs in one-hour, assembly-style sessions over three consecutive days, with a one-day booster session three months later. These sessions will ideally take place in the school auditorium or other assembly space (e.g., cafeteria, gym or multi-purpose room). Children in the intervention arm will receive the HHS curriculum and materials, while those in the control arm will receive standardized nutrition education. Students in both arms will complete a pre-test assessment on Day 1 and an immediate post-test at the end of Day 3 (Table 1). Three months after the 3-day program, children in both conditions will participate in a one-hour booster session, in which they will complete a delayed post-test. Parents complete the pre-assessment in-person or via telephone, depending on the method of recruitment, and the post-assessments via telephone.

#### Intervention condition

Hip Hop Stroke. Intervention modules were developed with learning objectives principles based on Bloom’s taxonomy, and designed for integration with the school’s calendar. They conform to NYS Education Department’s curriculum standards for health, physical education, and Family/Consumer Science. Modules incorporate multimedia and are assembled into curriculums that address either stroke treatment (recognition and action) or stroke prevention (primordial). Modules include a variety of songs, cartoon videos, comic books and a video game. Each module has been tailored culturally and contextually, taking into account the issue of health illiteracy and low overall literacy. The use of songs, cartoons, and storytelling is a strategy our group has employed for overcoming literacy barriers and enhancing engagement with content.

#### Day 1

Children will complete the baseline survey before being introduced to the topic of stroke, including the types of stroke, stroke localization, signs and symptoms, and F.A.S.T. acronym for rapid assessment and appropriate immediate response (call 911). Children will also view a child-centric cartoon developed for this program, “Stroke Ain’t No Joke,” which will reinforce the signs, symptoms and response, as well as teach them about the “clot buster” medication, t-PA.

Students will be sent home with an activity packet that includes a comic book and a DVD, containing the two 4-minute animated features that focus on stroke recognition and stroke prevention. The students will be instructed and encouraged to engage their parents in completing the activities with them. Students will be asked to return an activity page from the comic book on Days 2 and 3. Students will be awarded prizes if at least 50% return their activity pages, with additional prizes for a return rate of at least 75%. In addition, students can view the same program cartoons on our website (www.hiphopublichealth.org) by creating a login and utilizing a trackable password/personal identification number given to them by the research team to gain access. Children are also able to access our “clot buster” game online [32].

#### Day 2

No testing will occur on the second day. All of the concepts introduced on Day 1 will be reviewed, and children will learn about the risk factors that may increase the chance of having a stroke, including poor nutrition, hypertension, diabetes, obesity, smoking and lack of exercise. Students will also view a second cartoon, “Keep your Brain Healthy,” which tells the “child mediated health communication” story of a father who eats unhealthily, does not exercise, smokes and drinks alcohol until he has a stroke and is motivated by his son to change his lifestyle. A magnet highlighting stroke symptoms and response will be sent home with each child to reinforce stroke symptom learning.

#### Day 3

The health educators will briefly review the concepts taught over Days 1 and 2. Children will review the “Stroke Ain’t No Joke” and “Keep your Brain Healthy” cartoons for the second time in a discussion format. Students will be motivated once again to engage their parents with the health messaging and provided an opportunity to role-play communicating the health messages to their parents and calling 911. At the conclusion of the 3-day program, students will complete an immediate post-test. The students will then be provided with a cartoon greeting card highlighting the F.A.S.T. acronym to take home to their parents.

#### 3-month booster

Three months following the 3-day intervention, students will assemble for an unprompted, delayed post-test. After that, our health educators will deliver an educational booster session to the students that review stroke subtypes, signs and symptoms, and appropriate response. Children view the “Stroke Ain’t No Joke” and “Keep your Brain Healthy” cartoons again (total of 3 views of each cartoon over three months).

#### Attentional control condition

MyPyramid in Egypt. The program selected for the control arm, “MyPyramid in Egypt,” will address nutrition, physical activity and obesity education. This program was selected because nutrition, physical activity and wellness programs are now being incorporated into New York City public school curriculums as part of a legislative directive. The New York State “Healthy Schools Act” of 2007 requires school districts to establish “School Wellness Policies” that include a nutrition education curriculum and physical activity.

Trained facilitators will conduct “MyPyramid in Egypt” using Egyptian history as an entry point for the USDA’s MyPyramid nutrition program. Students will learn about MyPyramid and MyPlate across the 3-day one-hour-a-day program. The educators will be shown an edited music video for Michael Jackson’s Egyptian-themed video “Remember the Time.” Similar to the intervention group, control children will be provided with a take-home activity sheet on Day 1 to complete. Parallel pre and post-tests will be conducted with the control children, using the same testing sequence as the intervention group. Three months later, students will be assembled by program facilitators for an unprompted, delayed post-test and booster “MyPyramid in Egypt” session to mirror intervention procedures.

### Data collection and management

The surveys and data collection procedures will be the same for both MyPyramid and HHS. All study data collection is performed or supervised by trained RAs.

#### Parent data

All parents will complete the baseline assessment either in-person or via telephone. After the initial 3-day program with the students, parents will be called to complete the follow-up survey, consisting of the same baseline questions as well as two new questions about whether their children have shared any information from the program with them or shared any of the educational giveaways (e.g., comic book, DVD). The same survey will be administered three months later after the booster session.

#### Student data

Study measures will be obtained from the children during the assembly-style program via hand-held wireless devices. Prior to beginning the program, each child will be given a hand-held wireless keypad (ARS: Audience Response System) [33] and a registration card. They will be instructed to write down the unique numerical identifier from their keypad, along with their name, grade and gender, in order to match up the ARS responses to each student. Day 1 will commence with a survey on stroke knowledge, which will be repeated on Day 3 and at the three-month booster session.

#### School data

All school programs will be collectively rated by the research team after the three-day program, and reviewed again upon completion of the booster session. Schools will be rated for overall experience of the program and data collection as well as the research team’s perception of their own performance.

#### Data safety and monitoring board

To ensure the safety of participants and the validity and integrity of the data, a data and safety monitoring board (DSMB) has been established. The DSMB includes five senior investigators with expertise in stroke, biostatistics and clinical trials. The DSMB will perform the following activities: (1) review the research protocol and plans for data and safety monitoring, (2) evaluate the progress of the interventional trial, including periodic assessments of data quality and timeliness, participant recruitment, accrual and retention, participant risk versus benefit, performance of trial sites, and other factors that can affect study outcome, (3) make recommendations to the investigators concerning continuation or conclusion of the trial, and (4) protect the confidentiality of the trial data and the results of monitoring.

### Measures

#### Stroke symptoms and response knowledge

For both parents and children, we will assess pre- and post-test knowledge of stroke symptoms and recommended action (i.e., calling 911), including stroke localization (Where in the body does a stroke occur?), signs and symptoms of stroke, and appropriate response to a hypothetical stroke scenario. Knowledge of signs and symptoms will be derived from the widely used BRFSS stroke knowledge instrument [34] and assessed using seven Yes/No questions, five real symptoms and two distracters, including blurred vision, facial droop, slurred speech or confusion, imbalance and severe headache, versus chest pains and coughing hard. Knowledge of stroke prevention measures will be assessed by six Yes/No questions, five real and one distracter, that ask subjects about the relationship between stroke risk and fruit and vegetable consumption, exercising every day, smoking avoidance or cessation, exercising once-a-week, medication adherence, and adding salt to meals.

#### Self-efficacy

Children’s and parents’ self-efficacy to appropriately recognize and respond to stroke will be assessed at baseline, immediate post-test and the three-month follow-up. We will ask parents and children about their confidence related to being able to tell that someone is having a stroke, knowing what to do if someone is having a stroke, telling the 911 operator why they think that someone is having a stroke, and teaching parents or friends about stroke.

#### Barriers to calling 911

We will also assess parents’ barriers to calling 911 at baseline. Parents will be presented with seven hypothetical scenarios. Each scenario begins, “If I think that I am having a stroke, I would not call 911 because…” and then a different possibility is suggested. For example, he/she believes that an ambulance costs too much, personal religious beliefs prevent him/her from calling 911, or he/she knows someone who previously had a bad hospital experience.

#### Child-mediated communication

At immediate and delayed follow-up, we will assess whether the child has talked with one of the adults in the household about what he/she learned in school. Parents will be asked whether their children shared any information about the program as well as whether they brought home any activities from the program to complete with them at home.

#### Demographics

At baseline, parents will be asked to report their age, gender, number of adult residents living in their household, race/ ethnicity, education level, occupation, stroke experience, health literacy, telephone number, email address and home address.

### Sample size and power analysis

Primary analysis will focus on the change in adults’ knowledge of stroke symptoms and the recommended response. Based on our pilot data [30], we will use a conservative estimate of the intervention effect. We will assume a 4% baseline rate for knowledge of all five cardinal stroke symptoms plus chest pain as a distracter symptom, plus calling 911 in response to a hypothetical stroke-in-action scenario, and a 24% increase over this rate in the intervention arm.

Based on an observed 2% increase in knowledge of the five cardinal symptoms in one community as a result of non-targeted public education efforts over a six year period [35], we hypothesize a less than 2% change in control parents' knowledge. Children in the control condition will have poor baseline knowledge of stroke symptoms and will not receive any formal stroke education, so the chances of them communicating meaningful stroke information to their parents will be negligible. Parents in the control condition will likely get stroke information only from existing public education efforts in the form of television or radio outreach, and overlapping local stroke outreach efforts unknown to us.

Based on our assumptions and using a significance level of 0.05, a sample size of 860 parents from 22 schools (11 intervention; 11 control) will provide 90% power to detect a difference between the intervention and control conditions in the parents’ ability to name all five cardinal symptoms of stroke, plus 1 distracter, and appropriately calling 911. Given that we will randomize schools, rather than individual participants, the sample size calculations include adjustment for the cluster randomization scheme using an intra-class correlation coefficient of 0.10. Power for secondary hypotheses is addressed below.

### Data analysis plan

The approach to analysis of binary outcomes will be generalized estimating equations (GEE) with a logit link, a generalization of logistic regression that allows for clustering. In this case, as the randomization will be by school, the analysis procedures will account for the possibility of having multiple parents per child and repeated assessments per parent/child. For continuous outcomes, we will use mixed-effects models, a generalization of regression that accommodates clustering. Unless otherwise noted, all hypothesis testing will be two-sided.

#### Hypothesis 1

No differences in baseline knowledge will exist between the parents assigned to the intervention and control arms or between the children assigned to the intervention and control arms. A mixed-effects model will compare intervention and control arms with respect to parents’ and children’s baseline knowledge, taking into account the clustering of children within school and parents within children. The comparison will be expressed as the mean baseline knowledge difference and corresponding confidence interval. This analysis will utilize all children and parents included in the study at baseline.

#### Hypothesis 2

Children in the intervention arm will demonstrate greater knowledge concerning stroke symptom identification and response immediately after and at three months following the intervention compared to those in the control arm. GEE will compare children's knowledge of stroke symptom identification (coded as success/failure, answering all questions correctly) between the intervention and control arms 1-week following the intervention and at three months. Baseline knowledge will be included in the analysis as a covariate. The comparison will be expressed as an odds ratio with corresponding confidence interval. This analysis will utilize all children included in the study. Assuming that 27.3% of children in the intervention arm remember 100% the symptoms and zero percent in the control arm, there will be >99% power to detect a difference between the groups using a 5% type I error rate.

#### Hypothesis 3

Compared to students in the control condition, children in the intervention arm will be more likely to communicate stroke information to their parents (assessed at 1-week follow-up). GEE will be used to compare the probability that stroke information is communicated to children's parents, as assessed at 1-week of followup, between the intervention and control arms. The comparison will be expressed as an odds ratio with corresponding confidence interval. This analysis will utilize all children-parent dyads included in the study. Assuming that 30% of children in the intervention arm will pass on the stroke information and zero percent in the control arm, there will be >90% power to detect a difference between the groups using a 5% type I error rate.

#### Hypothesis 4

In homes in which such communication has been enacted, parents in schools assigned to the intervention arm will demonstrate greater ability to name the symptoms of stroke and appropriate action, compared to their baseline knowledge, at 1-week and at 3-months follow-up, compared to parents in the control arm. GEE will be used to compare the probability that parents remember the symptoms of stroke (coded as success/failure, answer all correctly) at 1-week and 3-months follow-up between the intervention and control arms. We will conduct a repeated measures analysis including the one-week and three-month follow-up time points in a single model and use contrasts to make the specific time point comparisons. The comparisons will be expressed as odds ratios with corresponding confidence intervals. This analysis will utilize approximately 30% of homes included in the study, the percentage in which we expect communication to be enacted. The power for the comparison at three months was discussed in the section on statistical power. Assuming the difference in increased knowledge is greater at the one-week follow-up time point than at three months, we will have at least 90% power to detect a difference between treatment groups at one-week of follow-up with a 5% type I error rate.

#### Mediation analyses

We will also include analyses to establish if the effect of intervention on adult knowledge is mediated by improvement in children's knowledge. In general, these analyses involve three regression equations: (1) regression of adult knowledge on the intervention, (2) regression of children's knowledge on the intervention, and (3) regression of adult knowledge on both the intervention and children's knowledge. For our analyses, we will need extensions of the classic single mediator model due to the multilevel structure of our data and categorical outcomes. The necessary modern mediation methods including these extensions are reviewed by MacKinnon et al. [36].

## Discussion

Since stroke patients activate 911 themselves only 2%–7% of the time [37], the emphasis of stroke education campaigns must include the general public. Stroke literate children, a captive audience in school systems, may add to the acute stroke witness/bystander pool that is mostly responsible for calling 911 in the event of a stroke and teach their parents and grandparents about stroke symptoms and their urgency, thereby improving overall household stroke literacy in high stroke risk communities.

Targeting children to intervene with their parents has been rarely and sporadically attempted in various content areas. These interventions have used traditional teaching methods that do not engage the children, and little success has been reported. In contrast, the HHS intervention was designed in collaboration with school-aged children, children’s education media experts, as well as public health experts, school principals and neurologists. As a result, not only is the targeting of children for this purpose an important innovation, but so is the careful development of materials designed to appeal to them. Moreover, we note that utilizing children as a “transmission vector” for carrying out interventions aimed at their parents has the potential to serve as the basis for interventions in any number of other areas, including medication adherence, healthy eating and weight loss and treatment of diabetes. Thus, the significance of the proposed trial addresses the public health problem under study-stroke symptom identification and response-as well as development and refinement of a more general model of intervention, especially in instances in which targeting the parents themselves has proved ineffectual.

Behavioral health interventions have often proven efficacious in trial, yet, by one criterion, must be considered failures as they rarely survive the funding period. There are several reasons for this, including high costs, poor cost-effectiveness and a lack of ability on the part of most researchers to translate their results into a commercially viable, or otherwise sustainable, intervention. In contrast, our intervention requires low maintenance costs for delivery given its media-heavy curriculum that can be downloaded from an online platform. It targets a captive audience of school children, removing the need for special arrangements to be made in the community for the delivery of the intervention. In addition, the principals of these schools are required by both state and federal law to conduct health education programs that include core measures addressed by HHS. Finally, state and federally designated stroke centers are required to provide community stroke education within their catchment area at least bi-annually and often suffer from a dearth of proven stroke education tools that they can utilize to meet these goals.

The HHS programs shows promise to address the challenges of community stroke education among high risk economically disadvantaged groups in the United States and improve prehospital delays to acute stroke treatment.

## Figures and Tables

**Table 1:** Program overview.

	3-Day program implementation			Follow-up dose
	Day 1 (60 minutes)	Day 2 (60 minutes)	Day 3 (60 minutes)	3-month booster (60 minutes)
**Hip Hop Stroke**	Pre-test evaluation of baseline stroke knowledge	Stroke Prevention and Risk Reduction module	Review of days 1 & 2	Review of key concepts
	Stroke Recognition module		Immediate post-test of baseline stroke knowledge	Delayed 3-month post-test evaluation of baseline stroke knowledge and healthy living techniques
	Introduction of home activities			
**MyPyramid**	Pre-test evaluation of baseline stroke knowledge	Physical activity module	Review of days 1 & 2	Review of key concepts
	Healthy eating module (based on USDA food pyramid)		Immediate post-test of baseline stroke knowledge	Delayed 3-month post-test evaluation of baseline stroke
